# Reliability of focused cardiac ultrasound by novice sonographer in preoperative anaesthetic assessment: an observational study

**DOI:** 10.1186/s12947-015-0039-y

**Published:** 2015-11-20

**Authors:** Pawel Andruszkiewicz, Dorota Sobczyk, Izabela Gorkiewicz-Kot, Ilona Kowalik, Remigiusz Gelo, Orest Stach

**Affiliations:** 2nd Department of Anaesthesiology and Intensive Care, Warsaw Medical University, ul. Banacha 1A, 02-097 Warsaw, Poland; The Department of Interventional Cardiology, John Paul 2nd Hospital, ul. Pradnicka 80, 31-202 Cracow, Poland; The Department of Noninvasive Cardiovascular Diagnostics, John Paul 2nd Hospital, ul. Pradnicka 80, 31-202 Cracow, Poland; Institute of Cardiology, ul. Spartanska 1, 02-637 Warsaw, Poland

**Keywords:** Anaesthesia, Cardiac ultrasound, Echocardiography, Point-of-care

## Abstract

**Background:**

Use of preoperative echocardiography may help to identify patients with increased cardiac risk, who may benefit from modification of perioperative plan. The objective of our study was to evaluate the reliability of preoperative focused cardiac ultrasound (FoCUS) performed by an anaesthetist with basic ultrasound training and its impact on patient’s management.

**Methods:**

The prospective observational study was conducted in 159 adult patients, scheduled for elective operations. Cardiac ultrasound was performed by one anaesthetist with a limited experience of FoCUS. A simple, mnemonic scheme was used for the final reporting of each study. The same scheme was used by a cardiologist who produced an independent report based on digital video loops stored in the machine memory. Anaesthetists in-charge made final perioperative plan.

Comparative analysis of anaesthetist and cardiologist performed ultrasound report was made. The incidence of modification of initial perioperative plan resulting from FoCUS report was analyzed.

**Results:**

The average time required to complete the examination was 182 s 95 % CI [173–190]. Images of quality adequate to answer all questions from the scheme were obtained in 97.5 % (155/159) of patients. There was strong agreement between the anaesthetist and the cardiologist in 97.8 % (2274/2325) of the examined categories. In two categories (global and regional left ventricle contractility impairment) statistically significant discrepancies between both diagnosticians were confirmed (p McNemar <0.04). When compared with the cardiologist’s assessment the agreement of the anesthetist’s diagnosis had sensitivity of 0.84, specificity 0.99, positive predictive value 0.78 and negative predictive value 0.99. Kappa statistics showed good agreement between both examining doctors (κ = 0.797). Based on ultrasound findings, the preliminary anaesthetic plan was changed in relation to 20.8 % (33/159) of patients.

**Conclusions:**

An anaesthetist with limited training in FoCUS can perform a reliable preoperative examination which alters the perioperative management.

## Background

Cardiac complications are the leading causes of morbidity and mortality in the perioperative period [1–3]. Patients with heart disease are at particularly high risk. Prior identification of such patients and the severity of their disease may help an anaesthetist to optimize management of that specific disease [4, 5]. Preoperative assessment includes looking at the medical history, performing a physical examination and analyzing various cardiac investigations’ results [6, 7]. These methods, however, have limited reliability in diagnosing significant cardiac pathologies [8].

Although echocardiography provides important information on anatomy and heart function, it is still performed in minority of patients [4, 6]. One of the reasons is a limited access to formal cardiology-based echocardiography because of long waiting times [9, 10]. Unavailability of this service at short notice may lead to last-minute cancellations in surgery.

A growing body of evidence shows that focused cardiac ultrasound, performed by primary-treating physicians, with relatively brief training, can significantly influence clinical management [11–13].

The term cardiac ultrasound has been introduced lately to emphasize the difference of limited examination and formal echocardiography with respect to technical requirements for equipment, expertise for image acquisition, proficiency in data analysis and interpretation [14–16].

Ultrasound can be a valuable supplement of physical examination during preoperative anaesthetic assessment. However it has been used for this purpose mainly by cardiac anaesthetists with expertise and formal training in transoesophageal (TOE) and transthoracic (TTE) echocardiography [10, 12, 17, 18].

One of the obstacles in routine implementation of this method into everyday practice is its complexity and thus the high risk of missing important pathologies [15, 16, 19]. Cardiac ultrasound based on a structured scheme may be particularly useful for novice sonographers during examination and final reporting [20–22].

The principal aim of this study was to evaluate the reliability of cardiac ultrasound performed by the anaesthetist – novice sonographer during preoperative patient assessment. Another aim was to evaluate an impact of ultrasound-based decisions on modification of patients’ management.

## Methods

The prospective observational study was performed in accordance with the Helsinki Declaration. Written, informed consent was obtained from all patients. The study was approved by the Institutional Ethical Committee (Ref. nr: KB/172/2014. Date of approval: 12 August 2014). The methodology followed the international guidelines for observational studies.

The study was conducted from 1 October to 31 December 2014, at the university hospital, where around 12,000 operations are performed annually.

### Patients

Inclusion criteria included consecutive patients aged > 18 years, scheduled for elective operations from a broad range of surgical specialties represented in the hospital (general surgery, vascular surgery, ENT, neurosurgery), if the anaesthetist performing FoCUS was available.

### Anaesthetist (first diagnostician) performing focused cardiac ultrasound

Prior to the study the participating anaesthetist completed training which met the Polish Society of Anaesthesiology and Intensive Care requirements for certified FoCUS operators. It comprised:

- A two-day, hands-on workshop covering imaging of four basic views (parasternal, long- and short- axis, apical four-chamber and subcostal)
- 50 FoCUS examinations (at least 20 supervised by expert sonographer)

Apart from the above experience, before commencing the study, the anaesthetist performed 50 unsupervised FoCUS examinations with feedback from an expert cardiologist, based on a review of recorded film loops. He also completed an introductory class (1.5-h) into the A-F mnemonic scheme (Table 1) of cardiac ultrasound, which included valvular lesions' assessment with color Doppler.

**Table 1:** Mnemonic A-F scheme of cardiac ultrasound (Sobczyk D, Andruszkiewicz P. (Eur J Anaesthesiol 2014; 31:505–506)

A	aorta	Proximal aortic diameter >4 cm?	Y	N
		Is dissection flap present?	Y	N
B	both ventricles	RV/LV > 1	Y	N
		D-sign	Y	N
C	contractility	Is LV global contractility impaired?	Y	N
		Regional wall motion abnormalities of LV?	Y	N
		Is RV contactility impaired?	Y	N
D	dimensions	LVEDD > 6 cm	Y	N
		RVEDD > 4,2 cm	Y	N
		LA antero-post dim >4,5 cm	Y	N
		RA major > 5,4 cm	Y	N
		RA minor >4,4 cm	Y	N
E	effusion	Is pericardial perfusion present?	Y	N
		Is pericardial tamponade present?	Y	N
		Is pleural effusion present?	Y	N
F	further abnormalities (non specified above)		Y	N

LA-left atrium, RA-right atrium, LV- left ventricle, RV-right ventricle, EDD-end diastolic diameter

The above prerequisites meet level 1 competence, minimum requirements for performing unsupervised point-of-care cardiac ultrasonography, recommended by World Interactive Network Focused on Critical Ultrasound (WINFOCUS) experts [23].

The examining anaesthetist was not involved in patients’ perioperative care and was blinded to details of patient history and physical assessment.

### Cardiologist (second diagnostician) - gold standard reference FoCUS assessment

During the second stage of the study, the cardiologist with certified expertise in echocardiography revived digital video loops stored in the memory of the ultrasound (US) machine using the same A-F mnemonic reporting pattern. The cardiologist’s interpretation of each assessed category was treated as the gold standard reference. Although the cardiologist had knowledge about the patient’s characteristics' data, she was blinded to the interpretation of the FoCUS, made by the anaesthetist.

All cases of poor image quality, resulting in questionable interpretation and ultrasound indications for modification of perioperative management, were documented.

The digital video loops analysis was made between two and five days after the FoCUS examination. Thus the cardiologist did not interfere with clinical decisions pertaining to patients.

### Anaesthetists in-charge of perioperative plan

Anaesthetists in-charge of study patients were staff anaesthetists with at least 6 years of experience. They were informed by the examiner (first diagnostician) about the ultrasound findings. They were responsible for the final perioperative management plan.

If the decision about modification plan was made on the basis of FoCUS results the anaesthetist in charge had to indicate on a dedicated data sheet one of three categories of planned actions: 1. cancellation of operation; 2. altered monitoring; 3. altered choice of drugs

### Equipment and data acquisition

FoCUS was carried out with a Sparq system (Philips Ultrasound, Bothell, WA, USA) with a 2–4 MHz sector transducer. All patients underwent FoCUS before the operation in a pre-anaesthesia room or in the patient’s ward. The anaesthetist was not supervised by the cardiologist either during the examination or interpretation phase. FoCUS was performed in the left lateral position in the following views: parasternal long- and short- axis, apical four-chamber (including color Doppler), subcostal four-chamber and short axis. All images acquired by the examining anaesthetist were stored as five second video-loops. An A-F simple mnemonic scheme was used for final reporting of the examination. In the scheme, each consecutive letter of the alphabet represents a particular anatomical structure or measurement of cardiac function (Table 1) [21]. In each assessed category, only one”yes” or “no” answer was chosen.

The duration of FoCUS examination was also documented.

### Comparative studies

In the final stage of the study, both FoCUS examiners’ (anaesthetist compared with cardiologist) reports were analyzed and compared by the independent researcher.

The same researcher analyzed decisions (made by anaesthetists in-charge) concerning modification of perioperative management.

The primary end-point of the study was: reliability control of cardiac ultrasound reports performed by the anaesthetist- novice sonographer, using a mnemonic examination scheme and duration of the cardiac ultrasound examination.

The secondary end-point was any modification in the original anaesthetic management plan, after cardiac ultrasound had been performed.

### Statistical analysis

Statistical analysis was performed with SAS statistical software (version 9.2, SAS Institute, Inc. Cary, NC, USA). Continuous variables are presented as mean and standard deviation (SD). Categorical variables are described as absolute numbers and percentages. Differences in baseline demographics, physical findings and medical history were assessed using Student’s *t*-test for continuous variables and the Pearson *χ*2 test or Fisher exact test for categorical variables. The diagnostic value of the anaesthetist examiner to detect echocardiographic abnormalities was calculated in terms of sensitivity, specificity and positive predictive values (PPV) and negative predictive values (NPV).

Sensitivity was defined as the number of true-positive results divided by the total number of patients with echocardiographic abnormalities indicated by the cardiologist. Specificity was defined as the number of true-negative results divided by the total number of patients without echocardiographic abnormalities.

Comparisons between the novice examiner (anaesthetist) and the specialist diagnostician were made with the McNemar test. The Cohen κ coefficient for agreement between them was calculated, to test the hypothesis that concordance was greater than chance alone.

A two-tailed p value <0.05 was considered significant.

## Results

159 patients were included in the study. Their characteristics are recorded in Table 2. Images of adequate quality to answer all questions of the mnemonic scheme were obtained in 155 out of 159 cases (97.5 %). Patients with positive findings in cardiac ultrasound were statistically more likely to suffer from hypertension, chronic obstructive airway disease (COPD), ischaemic heart disease and arrhythmia. The average time required to complete the FoCUS examination with the A-F mnemonic (Table 1) was 182 s 95 % CI [173–190].

**Table 2:** Baseline patient characteristics

	Grand total *N* = 155	CUS ‘–‘ *N* = 81 (52.3 %)	CUS’ + ‘ *N* = 74 (47.7 %)	*P*-value
Demographic				
Age [mean, (SD)]	57.1 (16.4)	49.4 (15.6)	65.5 (12.7)	<0.0001
Female sex [no. (%)]	83 (53.5)	47 (58.0)	36 (48.7)	0.2424
BMI (kg/m^2^) [mean, (SD)]	26.2 (5.2)	26.6 (5.5)	25.7 (5.0)	0.3210
Medical history [no. (%)]				
Hypertension	89 (57.4)	36 (44.4)	53 (71.6)	0.0006
Diabetes	13 (8.4)	6 (7.4)	7 (9.5)	0.6453
Renal failure	8 (5.2)	3 (3.7)	5 (6.8)	0.4801
Chronic obstructive pulmonary disease	15 (9.7)	3 (3.7)	12 (16.2)	0.0085
Ischaemic heart disease	35 (22.6)	6 (7.4)	29 (39.2)	<0.0001
Heart failure	7 (4.5)	2 (2.5)	5 (6.8)	0.2596
Valve disease [no. (%)]	6 (3.9)	0 (0)	6 (8.1)	0.0106
Arrhythmia [no. (%)]	30 (19.4)	8 (9.9)	22 (29.7)	0.0018
ASA grade [no. (%)]				
I	41 (26.4)	34 (42.0)	7 (9.5)	<0.0001
II	77 (49.7)	37 (45.7)	40 (54.0)	
III	37 (23.9)	10 (12.3)	27 (36.5)	
Surgical procedure [no. (%)]				
ENT	45 (29.0)	27 (33.3)	18 (24.3)	0.4660
General + vascular	100 (64.%)	49 (60.5)	51 (68.9)	
Neurosurgery	10 (6.5)	5 (6.2)	5 (6.8)	
Duration of examination (sec) [mean, (SD)]	181.8 (53.3)	167.7 (48.5)	197.3 (54.5)	0.0005

1. CUS ‘–‘no abnormalities found in cardiac ultrasound; 2. CUS ‘+’ abnormalities found by Anaesthetist and/ or Cardiologist

### Comparative analysis (anaesthetist compared with cardiologist) of the results of the FoCUS examination based on the A-F mnemonic scheme

In three [presence of aortic flap dissection; RV (right ventricle) > LV (left ventricle) and D-sign] out of 15 categories of the A-F mnemonic, no abnormality was detected by two examining physicians. In the remaining 12 categories either the anaesthetist or/ and the cardiologist pointed out various cardiac pathologies. A detailed description of the findings is shown in Table 3.

**Table 3:** Comparative incidence of the assessed categories of the A-F scheme. Analysis of compliance (Anaesthetist compared with Cardiologist)

A-F scheme category	Concordant answers		Non-concordant answers		Kappa	*P*(McNemar)	Sensitivity (Anaesthetist) [95 % CI]	Specificity (Anaesthetist)
	True positive	True negative	False negative	False positive				
Proximal aorta > 4 cm	7 (4.5 %)	146 (94.2 %)	2 (1.3 %)	0 (0 %)	0.8683	0.1573	77.8 % [45.3–93.7 %]	100 % [97.4–100 %]
LV global contractility impaired	4 (2.6 %)	143 (92.3 %)	1 (0.65 %)	7 (4.5 %)	0.7815	0.0339	80 % [37.6–96.4 %]	95.3 % [90.7–97.7 %]
LV regional wall motion abnormalities	6 (3.9 %)	140 (90.3 %)	0 (0 %)	9 (5.8 %)	0.5463	0.0027	100 % [61.0–100 %]	94 % [88.9–96.8 %]
LVEDD > 6 cm	1 (0.65 %)	152 (98.1 %)	0 (0 %)	2 (1.3 %)	0.4951	0.1573	100 % [20.7–100 %]	98.7 % [95.4–99.6 %]
RVEDD > 4,2 cm	4 (2.6 %)	148 (95.5 %)	1 (0.65 %)	2 (1.3 %)	0.7173	0.5637	80 % [37.6–96.4 %]	98.7 % [95.3 – 99.6 %]
LA diameter >4,5 cm	12 (7.7 %)	138 (89.0 %)	2 (1.3 %)	3 (1.9 %)	0.8098	0.6547	85.7 % [60.0–96.0 %]	97.9 % [93.9 –99.4 %]
RA major diameter	6 (3.9 %)	146 (94.2 %	3 (1.9 %)	0 (0 %)	0.7903	0.0833	66.7 % [35.4–91.0 %]	100 % [97.4–100 %]
RA minor diameter	9 (5.8 %)	141 (91.0 %)	4 (2.6 %)	1 (0.65 %)	0.7655	0.1797	69,2 % [38,9–89,6 %]	99,3 % [95,6 –100 %]
Pericardial fluid	4 (2.6 %)	150 (96.8 %)	1 (0.65 %)	0 (0 %)	0.8856	0.3157	80,0 % [29,9–98,9 %]	100 [96,9–100 %]
Pleural fluid	2 (1.3 %)	153 (98.7 %)	0 (0 %)	0 (0 %)	1.000	NA	100 % [29,9–100 %]	100 % [96,9–100 %]
Valvular lesions	39 (25.2 %)	109 (70.3 %)	2 (1.3 %)	5 (3.2 %)	0.8866	0.2568	88,6 % [74,6–95,7 %]	98,2 % [93,0–99,7 %]

LV- left ventricle, LA-left atrium, RV-right ventricle, RA-right atrium, EDD- end diastolic diameter

Differences between two examiners were statistically significant in evaluation of both global and regional contractility of the left ventricle (*p* = 0.03 and *p* = 0.003 respectively).

In cumulative analysis of all categories (*n* = 2325) of the A-F mnemonic there was an agreement in results between the anaesthetist and cardiologist in 97.8 % of the cases with kappa and p McNemar: 0.797 and 0.208 respectively (Table 4). When compared with the gold standard reference (the cardiologist’s assessment) the anaesthetist's diagnosis sensitivity was 0.84, specificity 0.99 with a PPV of 0.78 and NPV of 0.99.

**Table 4:** Cumulative analysis of compliance (Anaesthetist compared with Cardiologist) in all assessed categories of the A-F scheme

All categories		*n* = 2325 (100 %)
Concordant answers +	Anaesth+	108 (4.65 %)
	Cardiol +	
Concordant Answers -	Anaesth -	2166 (93.2 %)
	Cardiol -	
Non-concordant	Anaesth -	21 (0.9 %)
	Cardiol +	
Non-concordant	Anaesth +	30 (1.3 %)
	Cardiol -	
Kappa		0.7974
*P* (McNemar)		0.2076
Sensitivity (Anaesthetist) [95 % CI]		83.7 % [76.4 – 89.1 %]
Specificity (Anaesthetist) [95 % CI]		98.6 % [98.0 – 99.0 %]
Positive predictive value (PPV) [95 % CI]		78.3 %) [70.7–84.3 %]
Negative predictive value (NPV) [95 % CI]		99.0 % [98.5–99.4 %]

1. Anaesth + (pathology pointed by anaesthetist); 2. Anaesth- (no pathology pointed by anaesthetist); 3. Cardiol + (pathology pointed by cardiologist); 4. Cardiol - (no pathology pointed by cardiologist)

In 2166 out of 2325 (93.2 %) categories both examiners excluded any abnormalities.

In the remaining 159 (6.8 %) categories one of the examining physicians pointed out at least one abnormality.

### Analysis of decisions about modification of management (Figure 1)

**Fig. 1:**
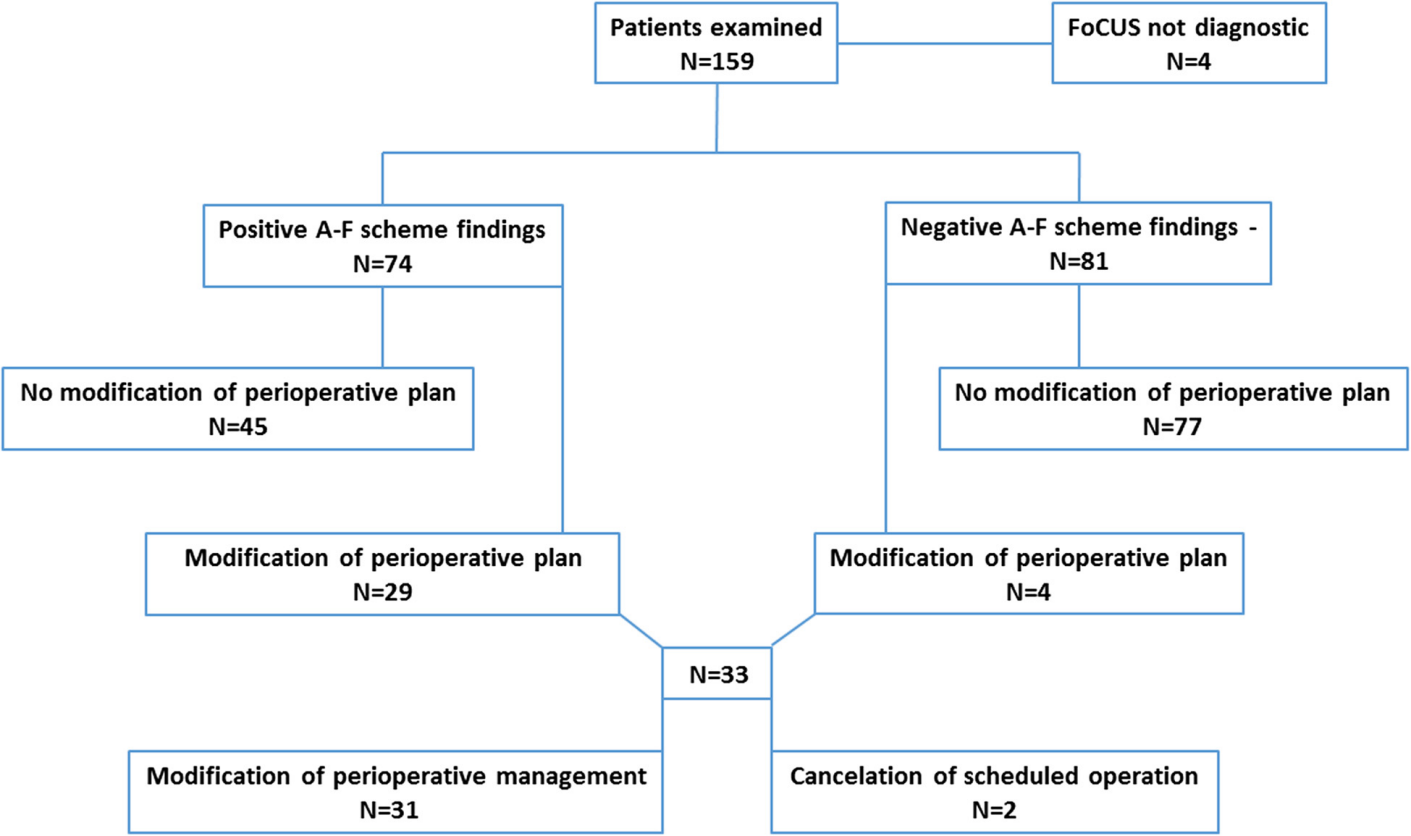
Modification of initial perioperative plan

In 33 out of 159 patients (20.8 %) anaesthetists in-charge of the study patients decided to alter initial perioperative plan on the ground of information based on the FoCUS report.

In two cases the anaesthetist in-charge decided on cancellation of the operation; in the remaining 31 cases initial anaesthetic management was altered (monitoring, choice of drugs). There were four patients in this group for whom the former plan was changed in spite of lack of ultrasound findings.

The reasons for modification of initial perioperative plan were as follows: impaired global (19/33 = 57.6 %) and regional (22/33 = 66.7 %) contractility of LV; valvular lesions (17/33 = 51.5 %); enlargement of left atrium (9/33 = 27.3 %); enlargement of right atrium (7/33 = 21.2 %); proximal aortic diameter enlargement (2/33 = 6.1 %)

## Discussion

The results of our study show that anaesthetist with limited training in FoCUS may reliably and quickly perform complex cardiac ultrasound examinations guided by a simple mnemonic scheme during preoperative visits. Ultrasound-assisted examination was a valuable source of information and had a decisive impact on modification of anaesthetic management in the perioperative period.

### Use of FoCUS during preoperative assessment

Satisfactory image quality was obtained by the anaesthetist performing FoCUS in 97.5 % of cases and this proves that the acquisition of the necessary manual skill requires relatively short training. This result is better than those presented in previous studies of novice sonographers who were primary - treating physicians working in cardiology, intensive care or emergency medicine settings [24, 25]. Higher rates of interpretable views- 98 -100 % were achieved by experienced cardiac anaesthetists, which can be explained by their competence in transoesophageal (TOE) and transthoracic echocardiography (TTE) [10, 12].

Good examination conditions may have had an impact on the views obtained. The FoCUS was an elective procedure performed on fully cooperative patients placed in an optimal position. Worse results are presented in studies where cardiac ultrasound was conducted in extremely demanding conditions of pre-hospital care [25, 26].

### Use of A-F mnemonic for FoCUS reporting

Compiling systematic reports of complex cardiac ultrasound is a demanding challenge for novice sonographers. Simplified FoCUS examination must be performed according to a standardized, but restricted, scanning protocol [15, 16]. In our study we assessed usefulness of the A-F mnemonic (Table 1), an easy-to-remember checklist scheme, which guided novice sonographer through the study [21]. Binary (yes/no) choice of answers enabled the making of a complete report in relation to all 155 patients with interpretable cardiac views. Short duration time of the FoCUS examination (mean 182 s) was in our opinion a result of clear construction of the A-F scheme. Other authors present longer (> 10 min) examination times of point-of-care cardiac examinations [12].

### Reliability of anaesthetist – performed FoCUS interpretation

The most demanding part of a point-of-care ultrasound is making a reliable interpretation of obtained images [27], which may have an impact on proper therapeutic decisions. The results of our study showed a high concordance between the findings of the anaesthetist performing the examination and the cardiologist assessing digital video loops (κ =0.797 and p McNemar = 0.208). Results in all categories showed good agreement with overall sensitivity of 0.84, specificity 0.99, PPV 0.78 and NPV 0.99 (Table 4). Other studies showed similar results comparing interpretation of the findings between the anaesthetist performing a focused examination and cardiologist conducting a formal echocardiography [10, 12].

It is noteworthy however, that contrary to our study in both cited articles anesthetists performing examination were “proficient in TTE and TOE”.

Good results presented in our study confirm that the clinician’s specialty mattered little, provided that competence and adherence to established examination scheme is assured [15, 27].

Although the results of most categories evaluated with the A-F mnemonic showed a good agreement between two examiners, there were two categories (global and regional LV contractility impairment) where statistically significant discrepancies were confirmed (p McNemar = 0.03 and 0.003 respectively). In most cases they resulted from under-estimation of contractile function by the anaesthetist. Over-estimation of LV function by novice sonographers is shown in other studies [11]. Estimation of contractility used in FoCUS is based entirely on visual assessment (“eyeballing”) and this may lead to a higher risk of subjectivity and calculation errors especially in a view of limited experience of our diagnostician. However other studies showed that novice sonographers, were able to estimate LV function with reasonable accuracy (κ = 0.61 - 0.72) compared with the accuracy of cardiologists [11, 28, 29].

The reason for suboptimal diagnostic accuracy, when assessing the diameters of LV and RV, can be explained by the impact on the results of the binary categorization. Some borderline measurements could have been on the wrong side of the cut-off limits and were responsible for some of the false positive and false negative results.

The most common abnormalities identified during FoCUS examination in our study were valvular lesions (46/155 = 29.7 %). This broad group of pathologies (included in F - further abnormalities category of the A-F mnemonic) encompassed both stenosis and regurgitation of examined cardiac valves. Severity of valvular lesion was not assessed by the examining physicians. Strong agreement between two examiners in assessment of this category (κ = 0.89) shows that basic evaluation of competency of cardiac valves is relatively easy. Similar results were presented in a study of patients with heart murmurs assessed by anaesthetist competent with TTE [10].

In spite of some differences in FoCUS reports between two examiners, no case of major pathology was missed.

### Modification of perioperative management

In our study, anaesthetists in-charge of the patients decided to change the initial perioperative management in 33 out of 159 (20.8 %) patients based on FoCUS findings (Fig. 1). There were four patients whose management was changed despite lack of detected abnormalities in the FoCUS examination. Two of them were living kidney donors, with ultrasound evidence of suboptimal volume-filling, confirmed by substantial respiratory collapsibility of the inferior vena cava. As a result of that finding, they received an extra bolus of intravenous fluids before organ harvesting. In two others, with a history of cardiac disease, the decision was made to step-down initially planned extended haemodynamic monitoring.

In two cases anaesthetists in-charge made decision to cancel operation on the ground of the FoCUS examination and referred patients to the cardiologist. In the first case undiagnosed severe impairment of the LV contractility was found. In the second case evidence of severe stenosis of aortic valve was detected. Both pathologies may be associated with increased risk of perioperative complications and may require preoperative intervention [1, 2, 10].

In our study the most common reason for modification of initial perioperative plan was impairment of global and regional contractility of the LV. On the other hand we showed statistically significant discrepancies between anaesthetist and cardiologist in this category. This may have led to unnecessary modification of anaesthetic plan including invasive monitoring.

Other studies presenting alteration of perioperative plan resulting from anaestetist-performed TTE, show higher frequency of changes. Preliminary anaesthetic management was changed in approximately half of patients for elective and emergency non-cardiac operations with suspected cardiac disease [10, 12, 17]. Patients’ characteristic with respect to cardiac disease may explain difference in approach to modification of anesthetic management.

It is noteworthy that positive FoCUS findings were statistically more likely to happen in patients with hypertension, COPD, ischaemic heart disease and arrhythmia. In our opinion cardiac ultrasound may have a decisive impact on the choice of a perioperative plan in this population.

The study has several limitations. Evaluation of all the patients by a single examiner may raise the question whether this person was still a novice at the end of the study. Recruiting of multiple novice examiners would also allow evaluation of the interobserver variability and reduce the risk of positive or negative bias. Unfortunately, at the beginning of the study, only one anaesthetist, with basic certified competence, was available in our Department. Cardiologist’s evaluation of each case was based on digital video loops, recorded by the anaesthetist, and limited information about the patient. We decided on such a course because we were expecting problems with the availability of the cardiologists during the preoperative visits. Bias in the decision-making process, concerning perioperative action plans was minimized by using a different anaesthetist to perform the US examinations from those responsible for anaesthesia in each case.

## Conclusions

The results of our study show that an anaesthetist, with limited training in FoCUS, may perform complex, accurate and quick examinations, guided by a simple A-F mnemonic scheme, during preoperative visits. Cardiac ultrasound was a valuable source of information and had a decisive impact on modification of anaesthetic perioperative management.

## Abbreviations

COPD: chronic obstructive airway disease
EDD: end diastolic diameter
FoCUS: focused cardiac ultrasound
LA: left atrium
LV: left ventricle
NPV: negative predictive value
PPV: positive predictive value
RA: right atrium
RV: right ventricle
TOE: transoesophageal echocardiography
TTE: transthoracic echocardiography
WINFOCUS: World Interactive Network Focused on Cardiac Ultrasound
